# UBQLN4 is an ATM substrate that stabilizes the anti‐apoptotic proteins BCL2A1 and BCL2L10 in mesothelioma

**DOI:** 10.1002/1878-0261.13058

**Published:** 2021-08-30

**Authors:** Fang Liu, RunSang Pan, HongYu Ding, LiLing Gu, Yun Yang, ChuanYin Li, YongJie Xu, Ronggui Hu, Hui Chen, XiangYan Zhang, YingJie Nie

**Affiliations:** ^1^ Medical College Guizhou University Guiyang China; ^2^ GuiYang Maternal and Child Hospital Guiyang China; ^3^ State Key Laboratory of Systems Biology CAS Center for Excellence in Molecular Cell Science Innovation Center for Cell Signaling Network Shanghai Institute of Biochemistry and Cell Biology Chinese Academy of Sciences Shanghai China; ^4^ Department of Rehabilitation Guizhou Provincial People’s Hospital Guiyang China; ^5^ NHC Key Laboratory of Pulmonary Immune‐related Diseases Guizhou Provincial People’s Hospital Guiyang China

**Keywords:** ATM, BCL2A1, BCL2L10, mesothelioma, UBQLN4

## Abstract

ATM serine/threonine kinase (ATM; previously known as ataxia‐telangiectasia mutated) plays a critical role in maintaining genomic stability and regulates multiple downstream pathways, such as DNA repair, cell cycle arrest, and apoptosis. As a serine/threonine kinase, ATM has an array of downstream phosphorylation substrates, including checkpoint effector checkpoint kinase 2 (CHK2). ATM inhibits cell cycle progression by phosphorylating and activating CHK2, which plays an important role in the formation and development of tumors and participates in DNA repair responses after double‐stranded DNA breaks. In this study, we used a recently developed mammalian functional genetic screening system to explore a series of ATM substrates and their role in DNA damage to enhance our understanding of the DNA damage response. Ubiquilin 4 (UBQLN4), which belongs to the ubiquilin family characterized by its ubiquitin‐like (UBL) and ubiquitin‐associated (UBA) domains, was identified as a new substrate for ATM. UBQLN4 is involved in various intracellular processes, such as autophagosome maturation, p21 regulation, and motor axon morphogenesis. However, the biological function of UBQLN4 remains to be elucidated. In this study, we not only identified UBQLN4 as a substrate for ATM, but also found that UBQLN4 interacts with and stabilizes the anti‐apoptotic proteins Bcl‐2‐related protein A1 (BCL2A1) and Bcl‐2‐like protein 10 (BCL2L10) and prevents mesothelioma cell apoptosis in response to DNA damage. These findings expand our understanding of the role of UBQLN4 in mesothelioma and provide new insights into potential mesothelioma treatments targeting substrates for ATM.

## Graphical abstract

ATM phosphorylates an array of substrates that play essential roles in DNA damage response. In this study, we used a functional genetic screening system to explore ATM substrates and identified ubiquilin 4 (UBQLN4) as a new substrate for ATM. Phosphorylated UBQLN4 prevented cytochrome c release and inhibited apoptosis in mesothelioma during DNA damage by stabilizing BCL2A1 and BCL2L10. These findings expand our understanding of the role of UBQLN4 in mesothelioma and provide a new insight into mesothelioma treatment by targeting ATM substrates.

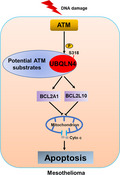

**Abbreviations**
53BP1: p53‐binding protein 1
ATM: ataxia‐telangiectasia mutation
BAP1: BRCA1‐associated protein 1 gene
BCL2A1: Bcl‐2‐related protein A1
BCL2L10: Bcl‐2‐like protein 10
BRCA1: breast cancer type 1
CHK2: checkpoint effector checkpoint kinase 2
CISP: cisplatin
COX IV: cytochrome c oxidase subunit 4
CPT: camptothecin
cyto c: cytochrome c
DOX: doxorubicin
DSB: DNA double‐stranded break
MDM2: murine double minute 2
MTX: methotrexate
NBS1: Nijmegen breakage syndrome protein 1
NSCLC: non‐small‐cell lung cancer
SMC1: structure of chromosome protein 1
TRIP12: thyroid receptor‐interacting protein 12
UBA: ubiquitin associated
UBL: ubiquitin‐like
UBQLN4: ubiquilin 4

## 1 Introduction

Maintaining genomic stability is crucial to all living organisms [1]. As a pivotal regulator of DNA damage response, ataxia‐telangiectasia mutation (ATM) kinase through checkpoint effector checkpoint kinase 2 (CHK2) pathway plays an important role in DNA damage, cell metabolism [2], and the cell cycle [3, 4]. ATM also maintains the structure of chromosome protein 1 (SMC1) [5], promoting DNA repair through activation of other DNA repair proteins such as p53‐binding protein 1 (53BP1) [6] and breast cancer type 1 (BRCA1) [7]. If the genome is damaged beyond repair, ATM induces programmed cell death by targeting histone H2AX [8], checkpoint protein 1 (MDC1) [9], Nijmegen breakage syndrome protein 1 (NBS1) [10], CHK2 [11], p53 [12], and murine double minute 2 (MDM2) [13]. Due to its multifaceted role in the DNA damage response, ATM dysfunction leads to several human diseases, such as breast cancer BRCA1/2 mutation‐related breast cancer [14] and p53 mutation‐related non‐small‐cell lung cancer (NSCLC) [15].

ATM phosphorylates an array of downstream substrates that play essential roles in DNA damage response. A comprehensive understanding of the interplay between these substrates in the DNA damage response is required to clarify the mechanism of DNA double‐stranded break (DSB) repair and will also provide new targets for the treatment of tumors. An array of ATM/ATR substrates and unique SQ/TQ phosphorylation domains has been identified using proteomic approaches [13, 16, 17]. DNA damage response substrate‐enriched domains including BRCT [18] and FHA [19] and histone interaction domains including TUDOR, CHROMO, and BROMO [20, 21] were also identified as substrates for ATM.

In this study, we screened a library of potential ATM substrates using a recently developed mammalian functional genetic screening system based on the use of GFP‐tagged shRNAs that were employed previously to explore the effect of drug sensitivity induced by specific gene knockdown on mouse Eμ‐Myc p19Arf^−/−^ lymphoma cells [22]. Using this approach, we identified many substrates for ATM, some of which were previously validated for DNA damage, such as UBR5, thyroid receptor‐interacting protein 12 (TRIP12) [23], lens epithelium‐derived growth factor p75 splice variant LEGDF [24], and BRCA1‐associated protein 1 gene (BAP1) [25]. This result confirmed the validity of this screening system for the identification of potential new ATM targets, and ubiquilin 4 (UBQLN4) was identified as a potential new substrate for ATM.

UBQLN4 is a member of the ubiquitin family characterized by the presence of ubiquitin‐like (UBL) and ubiquitin‐associated (UBA) domains. A previous study demonstrates that UBQLN4 interacts with ubiquitinated proteins and the proteasome via its UBA and UBL domains, respectively [26]. The ubiquitinated protein is then transported to the proteasome for degradation. Furthermore, recently studies suggested that UBQLN4 participates in the maturation of autophagosome regulation of p21 [27] and motor axon morphogenesis [28]. All these studies indicated that UBQLN4 plays an important role in variety cellular processes and may be a new target of precision therapy. However, the biological function of UBQLN4 in most tumors is still unclear. In this study, we not only identified UBQLN4 as new substrate for ATM, but also verified Ser318 as a major phosphorylation site following DNA damage. In a normal cell, pro‐ and anti‐apoptotic signals work together to maintain a balance between the life and death of the cell [29]. Our further study demonstrated that UBQLN4 acts as an anti‐apoptotic factor, which interacts with and stabilizes the anti‐apoptotic proteins BCL2A1 and BCL2L10, and regulates mesothelioma cell apoptosis in response to DNA damage. These findings expand our understanding of the role of UBQLN4 in mesothelioma and provide a new insight into mesothelioma treatment by targeting ATM substrates.

## 2 Materials and Methods

### 2.1 Cell culture and transfection

Eμ‐Myc p19^Arf−/−^ cell was kindly provided by Prof. Hai Jiang (Shanghai Institute of Biochemistry and Cell Biology). HEK293T, NCI‐H2452, and U2OS cell lines were purchased from the Chinese Academy of Sciences cell bank (Shanghai, China). B‐cell culture medium (45% Iscove’s modified Dulbecco medium and 45% Dulbecco’s modified Eagle’s medium (DMEM) supplemented with 5 μm β‐mercaptoethanol, 10% fetal bovine serum, and l‐glutamic acid) was used to culture Eμ‐Myc p19^Arf−/−^ cells. RPMI‐1640 (Gibco, Grant Island, NL, USA) containing 10% (v/v) fetal bovine serum (FBS, Biochrom, London, UK) was used to culture NCI‐H2452 cells. DMEM (Gibco) containing 10% (v/v) FBS, 100 U·mL^−1^ penicillin, and 100 μg·mL^−1^ streptomycin (Gibco) was used to culture HEK293T and U2OS cell lines. Polyethylenimine (Sigma, St. Louis, MO, USA) was used to transfect HEK293T cells, while U2OS cells were transfected using Lipofectamine 2000 (Invitrogen, Life Technologies, Carlsbad, CA, USA) according to the manufacturer’s instructions.

### 2.2 Plasmid constructs

Flag‐UBQLN4, HA‐BCL2A1, and HA‐BCL2L10 were cloned into the pCDNA3.0 vector for transiently transient transfection. BCL2A1 and BCL2L10 were cloned into pmCherry‐C1 vector, and UBQLN4 was cloned into the peGFP‐C1 vector for fluorescence colocalization experiments. The shRNA sequences were cloned into the lentiviral PLKO.1 vector for the ablation of UBQLN4. All restriction enzymes and ligases were purchased from NEB, and cloning was performed according to standard methods. shUBQLN4‐1 : TGCTGTTGACAGTGAGCGCCACCACTTTTGCAATCTTTAATAGTGAAGCCACAGATGTATTAAAGATTGCAAAAGTGGTGTTGCCTACTGCCTCGGA; shUBQLN4‐2 : TGCTGTTGACAGTGAGCGACCCAGAGGAAATTCGTGTGAATAGTGAAGCCACAGATGTATTCACACGAATTTCCTCTGGGCTGCCTACTGCCTCGGA; shBCL2A1 : GCCAGAACACTATTCAACCAATCAAGAGTTGGTTGAATAGTGTTCTGGCTTTTTT;shBCL2L10: GGCTTTTCTGTCATGCTTGTTTCAAGAGAACAAGCATGACAGAAAAGCCTTTTTT.

### 2.3 GFP‐based cell survival competition

Eμ‐Myc p19^Arf−/−^ cells were infected with a retrovirus expressing shRNA targeting a tumor suppressor gene and simultaneous expression of GFP. The infection efficiency of the virus was estimated to be between 20% and 40% based on the proportion of GFP‐positive cells. The cells were plated into 48‐well plates (10^6^ cells·well^−1^) and treated with different drugs at a lethal dose in the range of 80% and 90%. Half of the cells were removed from each experimental group every 24 h, and fresh medium was added. After 72 h, the viability of the drug‐treated and untreated cells was determined by flow cytometric analysis of propidium iodide (PI)‐labeled cells. The tolerance index to the relevant drug was then calculated. Cells in the untreated group, only 250 000 cells were plated, and 75% of the medium was replaced at every 24 h to avoid excessive growth of cells.

### 2.4 Calculation of drug resistance or sensitivity

The formula used to calculate drug resistance or sensitivity has been described previously [21]. We introduced the concept of the relative resistance index (RI) to better describe the drug sensitivity change caused by gene knockdown. The value of RI is defined as Y, where Y indicates that after treatment a mixture of GFP‐positive and GFP‐negative cells, the viability of infected (knockout) cells is changed Y‐fold in comparison with that of uninfected cells. For example, if one of the F‐uninfected cells survives after drug treatment, then Y cells survive after drug treatment in the F‐infected cells. If we define the total number cells (infected and uninfected) as T, and the proportion of GFP‐positive untreated cells as P1, the number of surviving cells that are not infected with the virus (F‐un) after drug treatment is F–un = T × (1–P1) × 1/F, and the number of surviving cells that are infected with the virus (F‐in) is F‐in = T × P1 × Y/F. Therefore, we can calculate the proportion of GFP‐positive cells in the surviving and drug‐treated population (P2) as P2 = (F–in)/(F–un + F–in). Therefore, Y = (P2–P1 × P2)/(P1–P1 × P2), using this equation to calculate RI values for each shRNA‐drug pair.

### 2.5 Cell cycle analyses

3 * 10^5^ NCI‐H2452 cells were plated into 12‐well plates, cultured overnight, and treated with 1 μm of camptothecin for 8 h, and then, the cells were collected and fixed overnight with 70% ethanol. Cells were then treated with 0.2% Triton X‐100, 100 μg·mL^−1^ RNase A, and 50 μg·mL^−1^ PI for 40 min and analyzed by flow cytometry.

### 2.6 Apoptosis analyses

3 * 10^5^ NCI‐H2452 cells were plated into 12‐well plates, and the related plasmids were overexpressed after overnight culture and treated with CPT. Cell apoptosis was evaluated by flow cytometric analysis of Annexin V staining using a 633 Apoptosis Detection Kit (Dojindo, AD11, Kyushu, Japan) according to the manufacturer’s instructions.

### 2.7 Colony formation assays

2 * 10^3^ NCI‐H2452 cells were seeded into 6‐well plates, and cells were treated with 0.2 μm, 0.5 μm, or 1 μm of camptothecin 24 h later. After another 24 h, the drug was removed and replaced with fresh medium and continued cultured at 37 °C with 5% CO2 for 10 days. Then, the cells were fixed with 4% paraformaldehyde, stained with 0.1% crystal violet for 30 min, and washed three times with PBS before being photographed.

### 2.8 Cell proliferation assays

NCI‐H2452 cells stably transfected with UBQLN4 shRNAs were plated in 96‐well plates (5 * 10^3^ cells·well^−1^ in triplicate) and treated with 4 μm, 2 μm, 1 μm, 0.5 μm, 0.25 μm, 0.125 μm, 0.0625 μm, 0.03125 μm, 0.015625 μm, or 0.007813 μm of camptothecin for 24 h. After 72 h, the number of viable cells was measured using the CCK8 method (Bimake, B34304). Briefly, CCK8 solutions were added, and the plates were incubated at 37 ℃ for 1 to 4 h, and then, the absorbance NCI‐H2452 cells were determined using a spectrophotometric plate reader (Thermo Fisher Scientific, Waltham, MA, USA).

### 2.9 Immunoprecipitation and immunoblotting

Cells expressing specific endogenous or exogenous proteins were lysed with immunoprecipitation (IP) buffer supplemented with a protease inhibitor cocktail (Bimake, B14001, Houston, TX, USA). After sonication, the cell debris was removed by centrifugation (13 000 ***g***, 10min), and the supernatant was aspirated and incubated with antiflag affinity gels (Sigma, A2220) at 4 °C for overnight. The beads were then washed and denatured at 95 °C for 10 min in 2 × SDS/PAGE loading buffer. Proteins were separated by SDS/PAGE and transferred to PVDF membranes (Bio‐Rad, 1620177, Guiyang, Guizhou, China). Membranes were then probed with antiflag (ProteinTech, 20543‐1‐AP, 1 : 1000, Guiyang, Guizhou, China) or anti‐HA (Sigma, H6908, 1 : 1000, St. Louis, MO, USA) antibodies. Secondary antibodies were labeled with HRP, and the signals were visualized using a Tanon 5200 Imaging System (Tanon, Shanghai, China).

Cells expressing the specific gene were lysed with 1 × SDS/PAGE loading buffer, denatured at 95 °C for 10 min, and subjected immunoblot analysis as described above. Detection was performed with the following antibodies : anti‐UBQLN4 (Santa Cruz, sc‐136560, 1 : 1000), anti‐pSQ/TQ (CST, 6966, 1 : 1000), anti‐BCL2A1 (ABclonal, A0134, 1 : 500), anti‐BCL2L10 (ProteinTech, 18114‐1‐AP, 1 : 500), antitubulin (ProteinTech, 66031‐1‐Ig, 1 : 1000), anticytochrome c(CST, 119405, 1 : 1000), anti‐COXIV (ProteinTech, 11242‐1‐AP, 1 : 1000).

### 2.10 Immunofluorescence analysis

2 * 10^5^ U2OS cells were transfected with peGFP‐UBQLN4 and pmCherry‐BCL2A1, or pmCherry‐BCL2L10 for 48 h, and then fixed with 4% paraformaldehyde. Cells were treated with 0.5% Triton X‐100 for 10 min at room temperature, and the nuclei were stained with DAPI for 5min. Image acquisition was performed with the Leica SP8, using identical confocal scan settings for each group.

### 2.11 Lentivirus production and infection

6 * 10^5^ HEK293T cells were seeded in 6‐well plates and co‐infected with pLKO.1‐shRNAs and psPAX2, pMD2.G. Cultivated at 37 °C supplemented with 5% CO2 for 72 h, then virus was harvested in 4 mL DMEM and used to infect NCI‐H2452 or 293T cells. After 24 h infection, successfully infected cells were screened with puromycin for 48–72 h.

### 2.12 Assay for cytochrome c release from mitochondria

Mitochondria‐free cytosol was prepared as previously described [30]. Briefly, 24 h after transfection and another 8 h treatment with CPT (1 μm), NCI‐H2452 cells were collected and washed twice with ice‐cold PBS, suspended in 100 μL extraction buffer (50 mm PIPES‐KOH, pH 7.4, 200 mm mannitol, 70 mm sucrose, 50 mm KCl, 5 mm EGTA, 2 mm MgCl2, 1 mm dithiothreitol, and protease inhibitors), and incubated on ice for 30 min. Cells were lysed by Dounce homogenization, and homogenates were centrifuged at 100 000 g for 15 min at 4 °C. Supernatants were harvested and analyzed by western blotting.

### 2.13 Immunohistochemistry (IHC) analysis of human tumor tissue array

Human mesothelioma tissue microarrays (purchased from Xi'an Elina Biotechnology Company) containing 30 mesothelioma cancer tissues and 10 adjacent normal tissues were analyzed by IHC using the VECTASTAIN ABC kit (Vector Laboratories, Burlingame, CA, USA). Rabbit primary antibodies for specific detection of UBQLN4 (ab106443, 1 : 300), BCL2A1 (ab45414, 1 : 200), and BCL2L10 (ab96625, 1 : 200) were purchased from Abcam. Sections were counterstained with hematoxylin and developed with diaminobenzidine. Immunohistochemical images were captured digitally. The expression of UBQLN4, BCL2A1, and BCL2L10 was measured by determining the integrated optical density sum of each photograph using image‐pro plus 5.1 software (Diagnostic Instruments, Sydney, Australia). The quantification of each sample was performed in 10 random fields (× 400) per case by two independent observers who were blinded to the clinical data. The study methodologies were approved by Guizhou University ethics committee.

### 2.14 Statistical analysis

All data were expressed as mean ± SEM unless stated otherwise. Student’s unpaired two‐tailed *t*‐test (95% confidence interval) was used to analyze data involving direct comparison of experimental and control groups. *P*‐values < 0.05 were considered to indicate statistical significance.

## 3 Results

### 3.1 Systematic analysis of ATM substrates by functional genetic screening

**Fig. 1:**
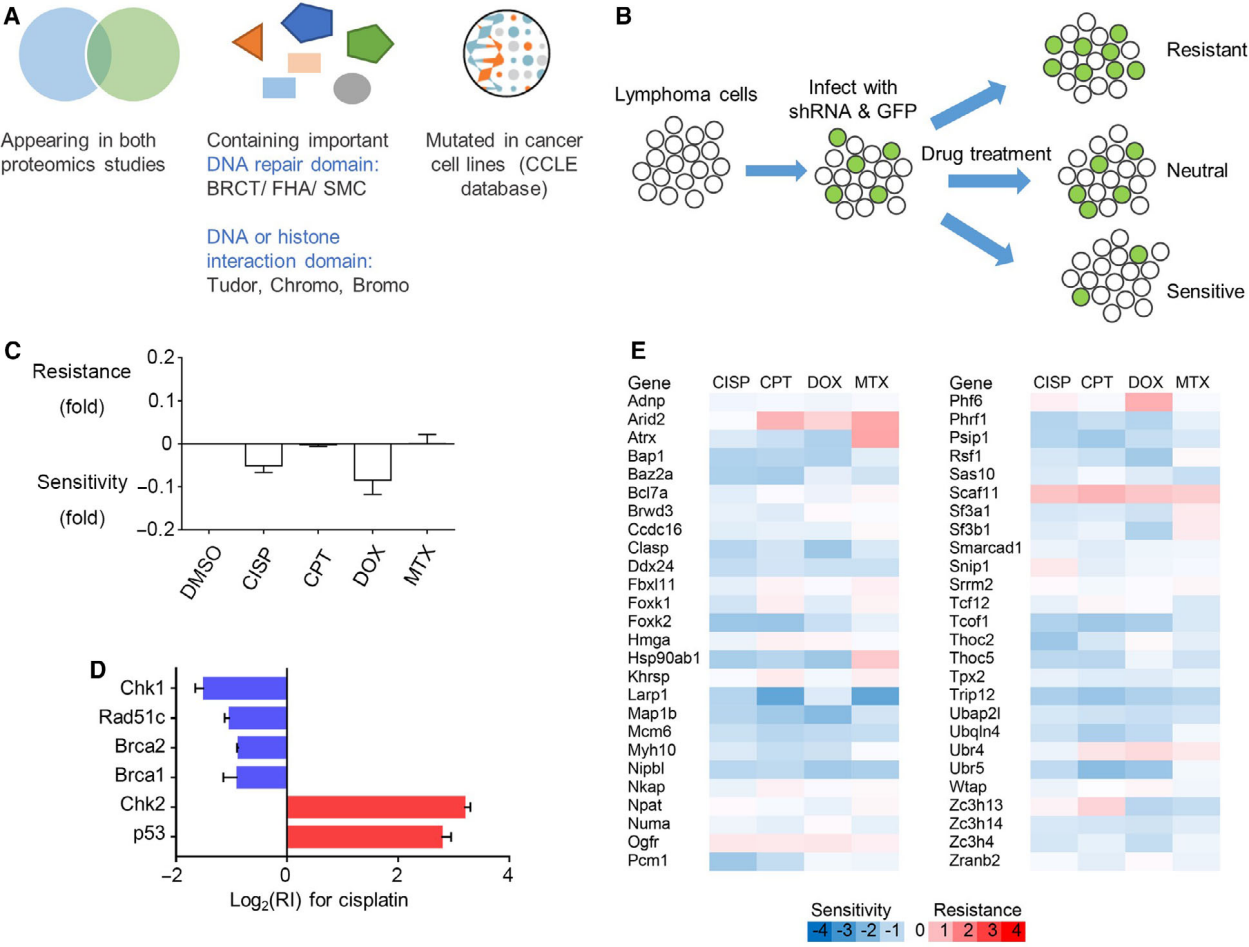
Identification of potential ATM substrate by functional screening. (A) Diagram of the ATM candidates. The potential ATM substrates were chosen from the overlap gene, which contains important DNA repair domains: BRCT/FHA/SMC, or DNA or histone interaction domains: Tudor, Chromo, Bromo, and mutated genes in cancer cell lines. (B) Cell survival assay based on GFP fluorescence. Eμ‐Myc p19^Arf−/−^ lymphoma cells were stably transduced with a recombinant retrovirus expressing shRNA and GFP. The shRNA‐medicated alterations in sensitivity to a specific DNA damage reagent were manifested as a change in the number of GFP‐positive cells. (C) Drug susceptibility to DNA damage drugs was detected in cells, CISP, CPT, DOX, and MTX. Values were normalized against vehicle controls. Data are presented in mean ± SEM, *n* = 3. (D) Proof‐of‐concept analysis showed that the depletion of known ATM targets including CHK1 and CHK2 altered the sensitivity to cisplatin. Data are presented in mean ± SEM, *n* = 3. (E) Heatmap summarizing how the depletion of potential ATM substrates leads to sensitivity (blue) or resistance (red) to a drug. For each gene, independent shRNAs exhibited similar resistance or sensitivity phenotypes.

We previously developed a functional genetic platform for the detection of genetic perturbations and altered drug sensitivities [22]. Briefly, a retrovirus encoding shRNA targeting a tumor suppressor gene and GFP were used to infect the Eμ‐Myc p19^Arf−/−^ cell line, which was then treated with DNA damaging drugs. If oncogene deletion renders cells more resistant to DNA damaging drugs, the proportion of GFP‐positive cells will increase. The platform was previously validated as a tool to study gene function and drug mechanisms [31, 32]. In this study, we used the platform to identify potential ATM substrates in response to drug‐induced DNA damage. Briefly, we selected a total of 52 cellular targets as potential candidates for ATM substrates based on previous publications and relevant databases containing important DNA repair domains (BRCT/FHA/SMC), or DNA or histone interaction domains (Tudor, Chromo, and Bromo) and mutated genes in cancer cell lines (Fig. 1A and Table S1). For each candidate, at least two independent shRNAs (Table S2) were pooled and used in the assay to limit the impact of off‐target effects. GFP‐encoded retroviruses that expressed these shRNAs were used to infect mouse Eμ‐Myc p19^Arf−/−^ lymphoma cells to knock down the expression of target genes. If the depletion of a specific gene increases the proportion of GFP‐positive cells in response to the drugs (Table S3), this gene is considered as a candidate for the DNA damage response. A reduction in GFP signal corresponds to drug sensitization, whereas increased GFP expression is interpreted as drug resistance (Fig. 1B).

For the control retrovirus vector did not alter drug sensitivity in our study, thus any changes in drug sensitivity were attributed to shRNA‐mediated gene silencing (Fig. 1C). Many advantages of this method make it beneficial for gene function studies of responses to DNA damage. Eμ‐Myc p19^Arf−/−^ cells have a relatively simple genetic landscape, and the data are highly reproducible since each sensitivity reading represents the results of tens of thousands of tumor suppressor‐deficient cells and collective survival of proficient cells. Finally, GFP‐negative candidate‐proficient cells serve as internal controls, which helps circumvent and normalizes sample‐to‐sample variability due to inaccurate cell passaging, cell seeding, different serum batches, medium evaporation, and other factors that could impact the consistency of the MTT assays.

As a proof of concept, we first evaluated the correlation between known tumor suppressor genes and drug sensitivity. It is well documented that deficiency in CHK1, RAD51D, and BRCA1/2 [14, 33] leads to cisplatin hypersensitivity, while CHK2 and p53 deficiency alters cisplatin resistance [34]. As expected, all five of the anticipated cisplatin sensitivity phenotypes were confirmed in our study (Fig. 1D). These results were consistent with clinical observations and validated the accuracy of our methods. After the proof‐of‐concept and validation studies, we then further employed this method to screen substrates for ATM in 52 candidates as described.

### 3.2 Confirmation of UBQLN4 as an ATM substrate that alters the cellular response to DNA damaging agents

**Fig. 2:**
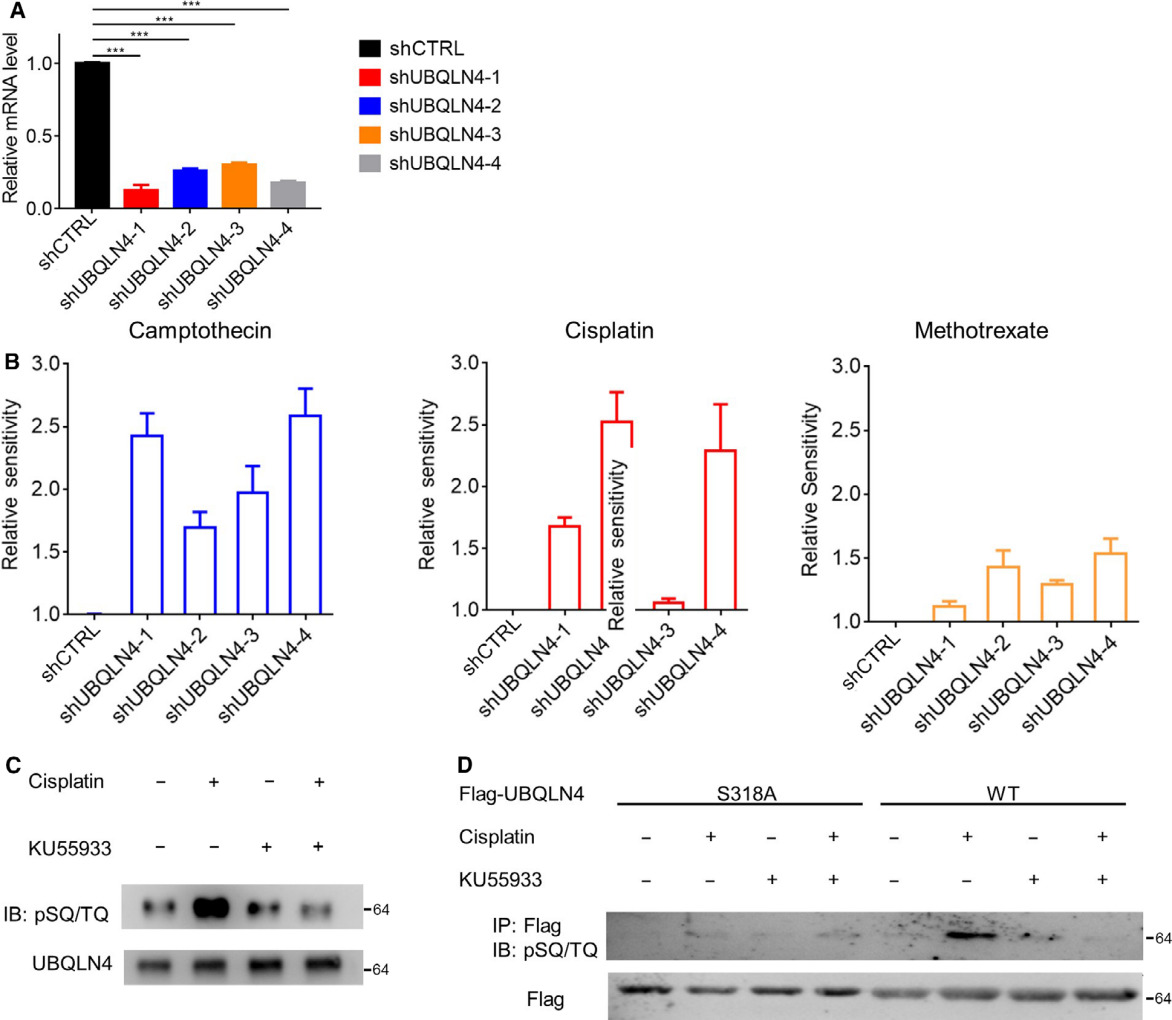
UBQLN4 was identified as an ATM pathway substrate. (A) The knockdown efficiency of UBQLN4 in Eμ‐Myc p19^Arf−/−^ cells was analyzed by qPCR. Data are presented in mean ± SEM, *n* = 3. Significance was determined by Student’s *t*‐test. ***P < 0.001. (B) Effect of UBQLN4 knockdown on Eμ‐Myc p19^Arf−/−^ cell sensitivity to DNA damage drugs (cisplatin, camptothecin, and methotrexate). Data are presented in mean ± SEM, *n* = 3. (C) HEK293T cells were treated with cisplatin and the ATM inhibitor KU55933 for 12 h. Endogenous UBQLN4 and ATM phosphorylation substrate‐specific antibodies (pSQ/TQ) were assessed, *n* = 3. (D) Wild‐type UBQLN4 or its S318A mutant was overexpressed in HEK293T cells. After 12 h of treatment with cisplatin and KU55933, UBQLN4 was immunoprecipitated with flag beads and subjected to immunoblotting analysis, *n* = 3.

The functional genetic screening results were analyzed using a previously developed calculation method [22] as described in the Experimental Procedures section. The best signal obtained from three shRNAs of the 52 potential ATM substrates screened is shown in Fig. 1E and Table S1. As a potential ATM substrate, UBQLN4 was chosen for further study. Our study demonstrated that the silencing of UBQLN4 resulting in significantly enhanced sensitivity to several DNA damaging agents (Fig. 2A–B). Endogenous UBQLN4 was phosphorylated in response to cisplatin, which was reduced by the ATM inhibitor KU55933, suggesting that the pSQ/TQ sites of UBQLN4 are ATM targets (Fig. 2C). Exanimate the full wild‐type UBQLN4 protein sequence to further map the specific residues phosphorylated revealed the serine residue at position 318 as the most likely candidate. Indeed, the mutated UBQLN4 (S318A) was no longer phosphorylated in response to DNA damage (Fig. 2D). These results confirmed that UBQLN4 is a new substrate for ATM, and Ser318 is a major phosphorylation site in response to DNA damage.

### 3.3 UBQLN4 is highly expressed in mesothelioma

**Fig. 3:**
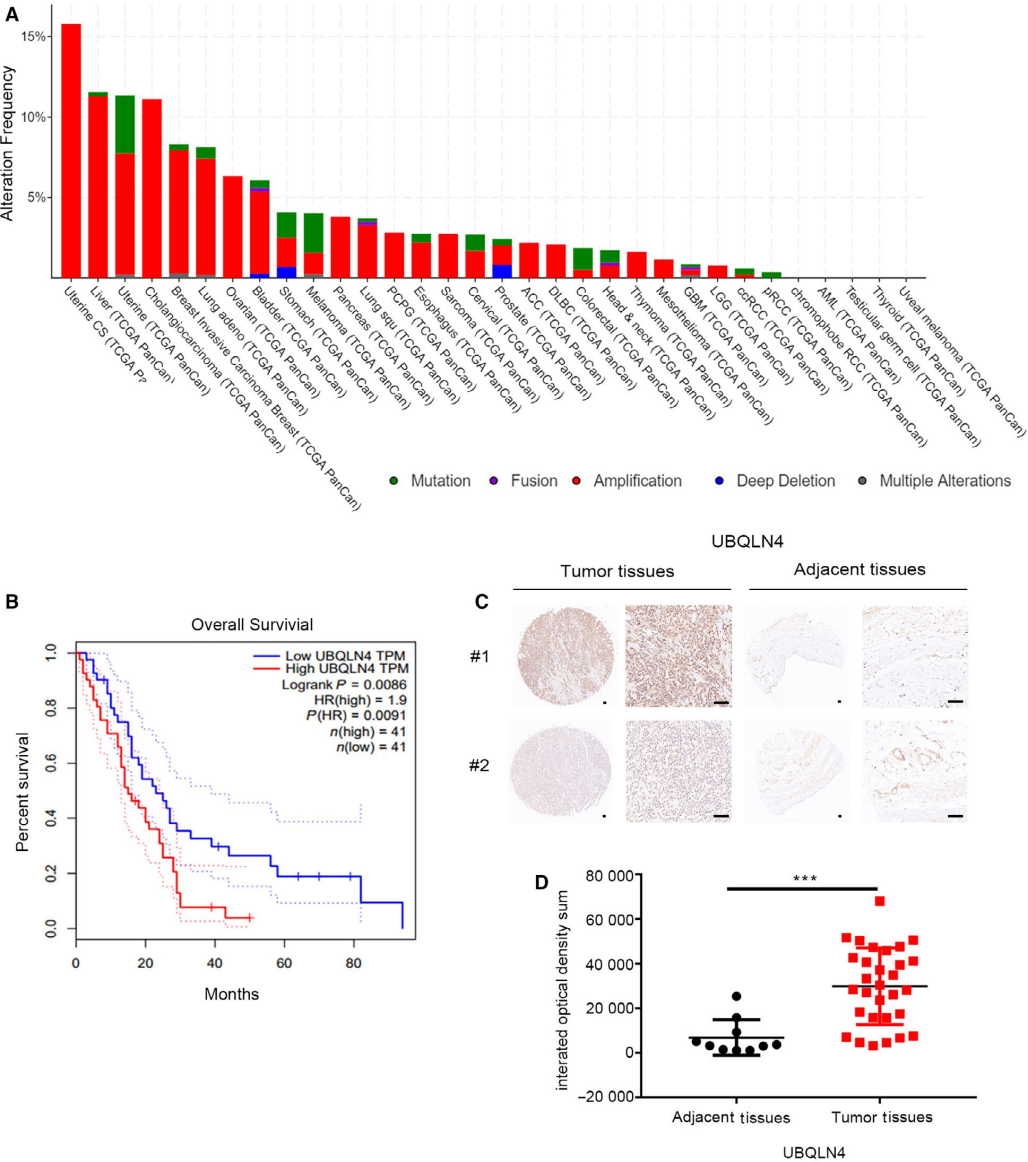
UBQLN4 is overexpressed in mesothelioma. (A) The X‐axis represents different tumor types, and the Y‐axis represents the alteration frequency, the red part represents amplification, the green part represents mutation, and the blue part represents deletion. (B) Correlation between UBQLN4 expression in mesothelioma and survival. GEPIA program from TCGA MESO datasets was used to construct Kaplan–Meier survival curves. Blue and red curves denote low‐ and high‐risk groups, respectively. X‐axis indicates the survival in months, and the Y‐axis represents the percentage survival. (C) Representative images of immunohistochemical staining of UBQLN4 in tumor tissues (*n* = 30) and adjacent tissues (*n* = 10). Scale bar, 100 μm. (D) The integrated optical density sum of UBQLN4 in tumor tissues (*n* = 30) and adjacent tissues (*n* = 10). Significance was determined by Student’s *t*‐test. Data are presented in mean ± SEM, ***P < 0.001.

According to the data from TCGA database, UBQLN4 is amplified in many cancers, the red part represents amplification, the green part represents mutation, and the blue part represents deletion (Fig. 3A). Survival analysis of the TCGA mesothelioma cancer database using GEPIA suggested that cancer patients with low levels of UBQLN4 survived significantly longer than those with high levels of UBQLN4 (Fig. 3B). In addition to the elevated gene expression, immunohistochemistry (IHC) analysis of human mesothelioma tissue arrays containing 30 mesothelioma tissues and 10 adjacent normal tissues showed that UBQLN4 expression was also significantly increased at the protein level. The detailed clinical data for these patients are shown in Table S4, and two representative images are shown in Fig. 3C. Stronger UBQLN4 staining was observed in the mesothelioma tissues compared with that of adjacent tissues (Fig. 3D). Collectively, these data suggested that UBQLN4 upregulation may be associated with development or prognosis of mesotheliomas.

### 3.4 UBQLN4 regulates mesothelioma cell apoptosis in response to DNA damage

**Fig. 4:**
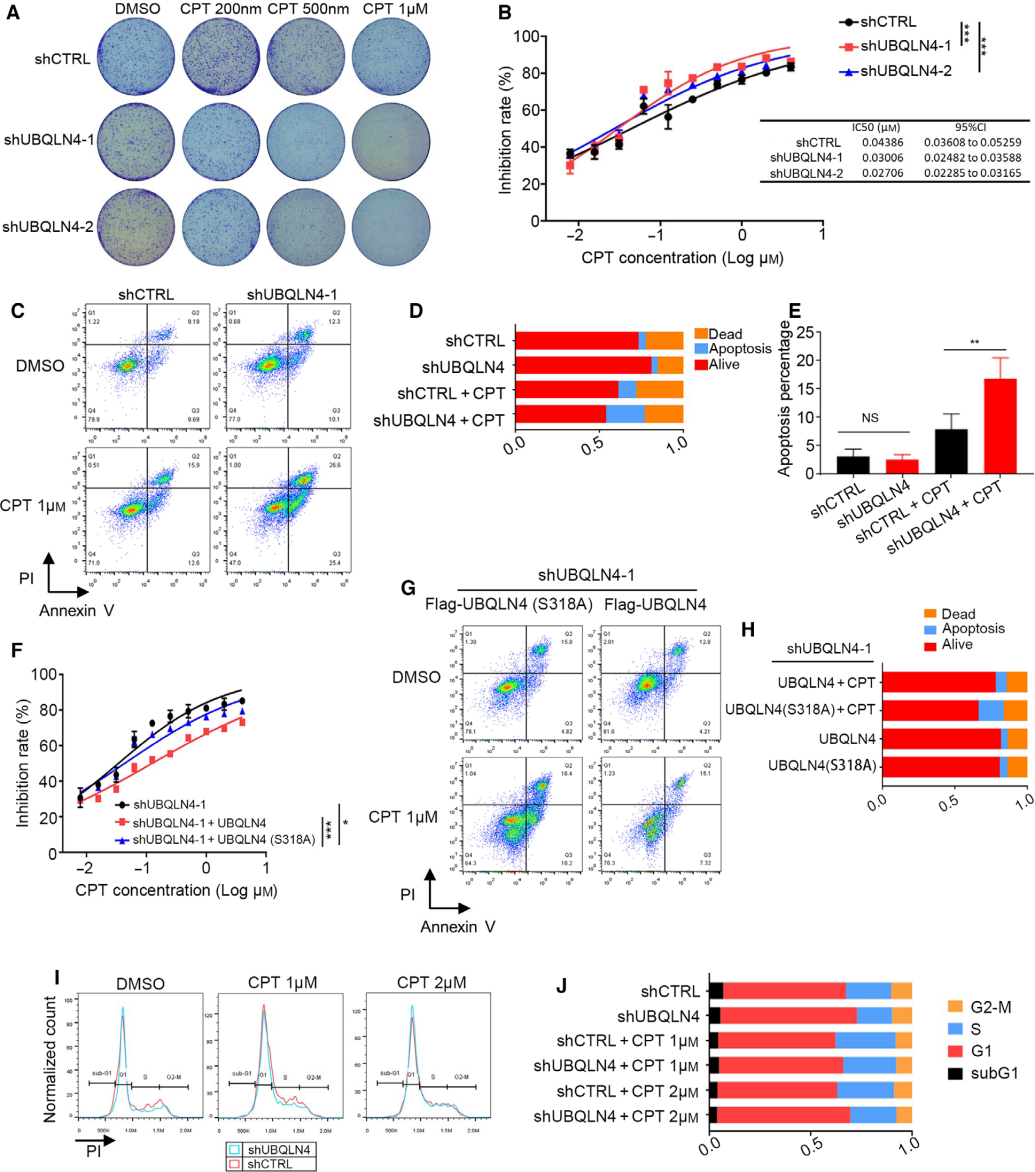
UBQLN4 regulates apoptosis in response to DNA damage. (A) UBQLN4 depletion sensitizes human mesothelioma NCI‐H2452 cells to DNA damaging drugs. Cell colonies were grown for 10 days after treating with CPT for 24 h, *n* = 3. (B) CCK8 assays were used to assess the viability of NCI‐H2452 cells following treated with CPT for 24 h. Significance was determined by one‐way ANOVA. Data are presented in mean ± SEM, *n* = 3, ***P < 0.001. (C–E) UBQLN4‐depleted NCI‐H2452 cells undergo increased levels of apoptosis in response to CPT (1 μm, 8 h). Significance was determined by Student’s *t*‐test. Data are presented in mean ± SEM, *n* = 3, **P < 0.01. (F) CCK8 assays were used to assess the viability of NCI‐H2452 cells following treated with CPT for 24 h. Significance was determined by one‐way ANOVA. Data are presented in mean ± SEM, *n* = 3, ***P < 0.001. *P < 0.05. (G‐H) UBQLN4 or UBQLN4(S318A) were overexpressed in UBQLN4 knockdown NCI‐H2452 cells, and levels of the apoptosis were evaluated by flow cytometric analysis in response to CPT (1 μm, 8 h). Data are presented in mean ± SEM, *n* = 3. (I–J) UBQLN4 silencing does not alter the cell cycle status of NCI‐H2452 cells treated with CPT (1 μm, 8 h). Data are presented in mean ± SEM, *n* = 3.

The colony formation and cell viability assays of human mesothelioma NCI‐H2452 cells indicated that UBQLN4‐silencing inhibited mesothelioma cell colony formation and significantly enhanced the mesothelioma cell sensitivity to DNA damaging drugs (Fig. 4A–B). Subsequently, Annexin V/PI staining assays showed a higher rate of apoptosis in UBQLN4‐deficient cells than that in control cells, suggesting that UBQLN4 regulates apoptosis in response to DNA damage (Fig. 4C–E). Furthermore, compared with UBQLN4‐S318A, overexpression wild‐type UBQLN4 significantly enhanced drug resistance to CPT treatment (Fig. 4F). The apoptosis induced by knockdown UBQLN4 could be restored by overexpression of UBQLN4‐WT, while UBQLN4‐S318A could not (Fig. 4G‐H). Flow cytometry analysis revealed that UBQLN4 silencing had no effects on cell cycle progression (Fig. 4I–J). Taken together, our data suggest that ATM‐mediated phosphorylation of UBQLN4 at Ser318 may regulate mesothelioma cell apoptosis in response to DNA damage.

### 3.5 UBQLN4 interacts with and stabilizes the anti‐apoptotic proteins BCL2A1 and BCL2L10

**Fig. 5:**
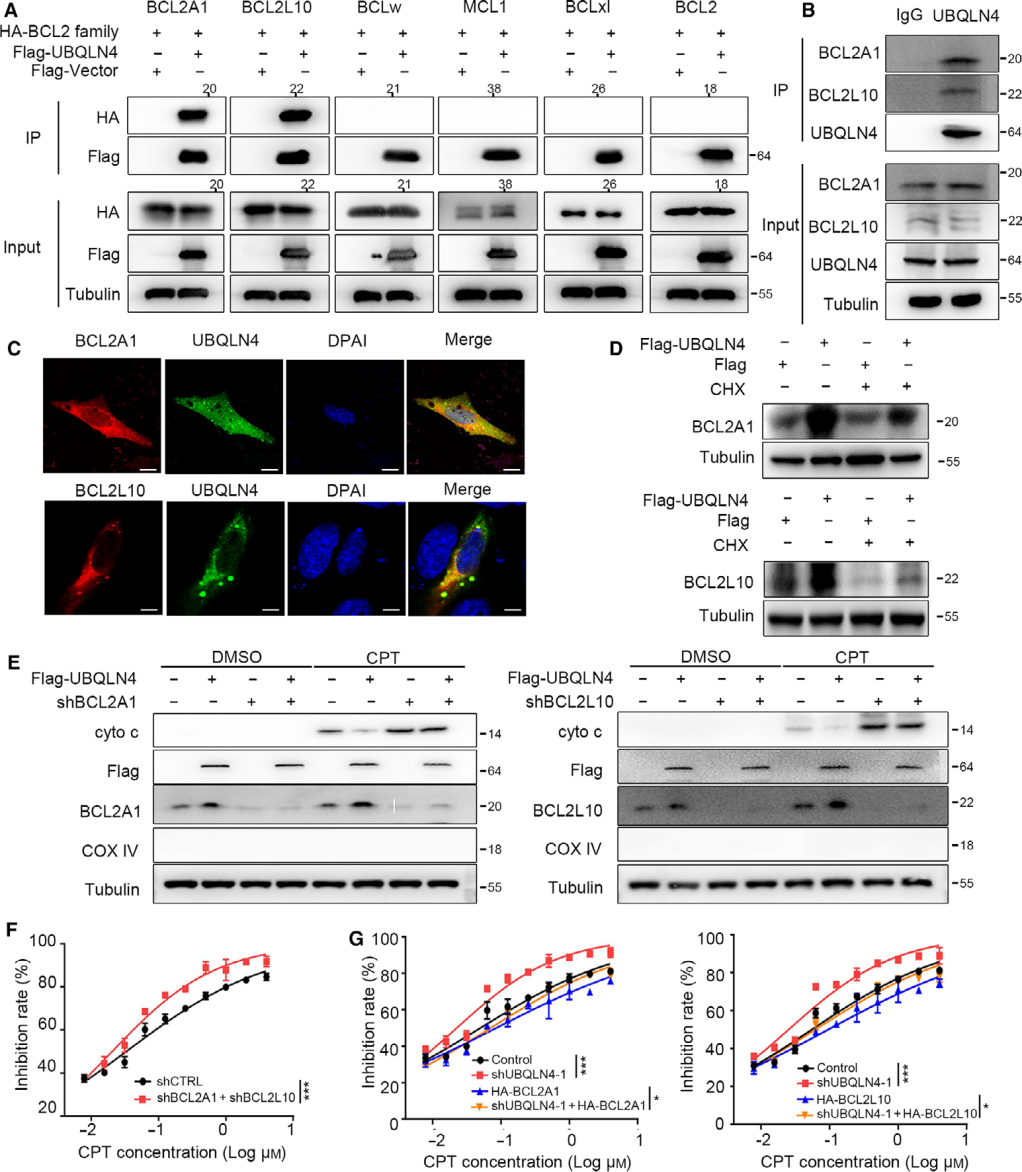
UBQLN4 interacts with and stabilizes BCL2A1/BCL2L10. (A) Co‐immunoprecipitation assays showing the interaction between ectopic UBQLN4 and BCL2A1, or BCL2L10, *n* = 3. (B) Co‐immunoprecipitation assay showed that endogenous UBQLN4 and BCL2A1 or BCL2L10 formed a complex, *n* = 3. (C) UBQLN4 colocalizes with BCL2A1 and BCL2L10. U2OS cells were cotransfected with pEGFP‐UBQLN4 and BCL2A1‐RFP, or BCL2L10‐RFP. Scale bar, 10 μm, *n* = 3. (D) UBQLN4 overexpression stabilizes BCL2A1 and BCL2L10. HEK293T cells were transfected with empty vectors or vectors expressing UBQLN4, and treated with CHX (100 μg·mL^−1^) for 12 h, *n* = 3. (E) The cyto3 was upregulated in NCI‐H2452 cells with BCL2A1 or BCL2L10 knockdown. After cell isolation, mitochondria‐free cytosol was collected for immunoblot with indicted antibodies, *n* = 3. (F) BCL2A1 and BCL2L10 were knocked down in mesothelioma cells and treated with CPT for 24 h. The cell viability was detected by CCK8 assay. Significance was determined by one‐way ANOVA. Data are presented in mean ± SEM. ***P < 0.001. (G) CCK8 assays were used to assess the viability of NCI‐H2452 cells following treated with CPT for 24 h. Significance was determined by one‐way ANOVA. Data are presented in mean ± SEM, *n* = 3, ***P < 0.001. *P < 0.05.

We next explored the role of UBQLN4 during apoptosis induction. UBQLN1, which is similar to UBQLN4, has been shown to interact with one of the BCL2 family members, BCL2L10, regulating its ubiquitination, intracellular localization, and stability. Therefore, we hypothesized that UBQLN4 might also interact with the members of BCL2 family. To test this hypothesis, we performed co‐immunoprecipitation analysis of UBQLN4 with six BCL2 family members. Flag‐tagged UBQLN4 and HA‐tagged BCL2, BCL2L10, BCLxL, BCLw, MCL1, and BCL2A1 were generated and cotransfected into HEK293T cells for co‐immunoprecipitation analysis. Cotransfection of UBQLN4 with individual BCL2 family members showed that UBQLN4 only interacted with BCL2A1 and BCL2L10, but not to other members of the BCL2 family (Fig. 5A). In addition, the endogenous UBQLN4 also could interact with the endogenous BCL2A1 and BCL2L10 (Fig. 5B). These interactions were further confirmed by colocalization assays using the fluorescent fusion proteins peGFP‐UBQLN4, and pmCherry‐BCL2A1 or pmCherry‐BCL2L10 expressed in U2OS cells (Fig. 5C). Next, the mechanism of UBQLN4 regulates BCL2A1 and BCL2L10 was investigated, and we found that overexpression of UBQLN4 in 293T cells led to an increase in BCL2A1 and BCL2L10 expression, suggesting that UBQLN4 can stabilize BCL2A1 and BCL2L10 (Fig. 5D). It is reported that the BCL2 family can inhibit mitochondrial outer membrane permeability (MOMP) and play an anti‐apoptotic effect [35, 36]. Therefore, we further investigated the anti‐apoptotic mechanisms of BCL2A1 and BCL2L10 in mesothelioma and found that UBQLN4 could prevent MOMP in mesothelioma cells with CPT treatment. Knockdown of BCL2A1 and BCL2L10 both could increase the MOMP and only partially rescue the effect of UBQLN4 treated with CPT (Fig. 5E). Besides, we found that the depletion of BCL2A1 and BCL2L10 could enhance the sensitivity to the treatment of CPT in mesothelioma cells (Fig. 5F). Furthermore, overexpression of BCL2A1 or BCL2L10 could rescue the effect of UBQLN4 depletion under the treatment of CPT (Fig. 5G). These results suggested that UBQLN4 regulates the apoptosis of cells by stabilizing BCL2A1 and BCL2L10.

### 3.6 BCL2A1 and BCL2L10 are highly expressed in mesothelioma which correlates with the expression of UBQLN4

**Fig. 6:**
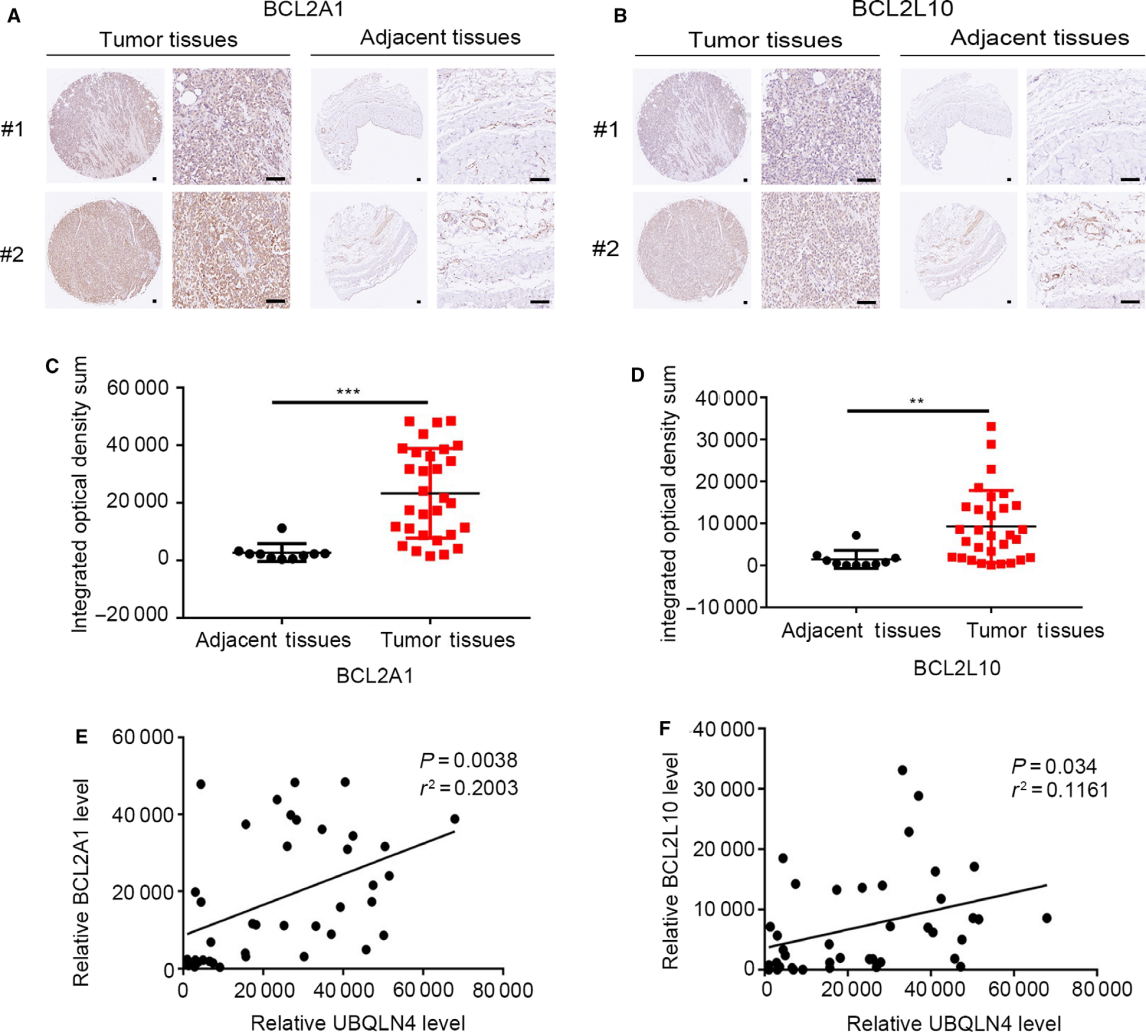
BCL2A1 and BCL2L10 are highly expressed in mesothelioma, and their expressions correlate with UBQLN4 level. (A) Immunohistochemistry shows the staining of BCL2A1 in tumor tissues (*n* = 30) and its adjacent tissues (*n* = 10). Scale bar, 100 μm. (B) Immunohistochemistry shows the staining of BCL2L10 in tumor tissues (*n* = 30) and its adjacent tissues (*n* = 10). Scale bar, 100 μm. (C–D) Statistics integrated optical density sum BCL2A1 (C) and BCL2L10 (D) staining in human mesothelioma tissues. Significance was determined by Student’s *t*‐test. Data are presented in mean ± SD, **P < 0.01. ***P < 0.001 (E–F). A correlation between UBQLN4 and BCL2A1 (E) or BCL2L10 (F) in mesothelium tissues (*n* = 40) was identified by Pearson's *r*. **P < 0.01, * *P* < 0.05.

Immunohistochemistry assays indicated that BCL2A1 and BCL2L10 are highly expressed in mesothelioma. As shown in Fig. 6A and Fig. 6B, much stronger staining for BCL2A1(A) or BCL2L10 (B) was observed in the tumors (left) compared with that in adjacent tissues (right). The expression of BCL2A1 and BCL2L10 was measured by determining the integrated optical density of each photograph as in Fig. 6C (BCL2A1) and Fig. 6D (BCL2L10). All three proteins, UBQLN4, BCL2A1, and BCL2L10, were highly expressed in all cases of mesothelioma tissues. The expression of UBQLN4 with BCL2A1 and BCL2L10 showed a positive correlation (Fig. 6E and 6F).

## 4 Discussion

In this study, we used a recently developed mammalian functional genetic screening system to explore new ATM substrates in a total of 52 cellular targets as potential candidates. We found UBQLN4 as a new ATM substrate, which was upregulated in mesothelioma. High level of UBQLN4 led to poorer prognosis than those with low levels of UBQLN4. Mechanically, UBQLN4 regulates mesothelioma cell apoptosis in response to DNA damage by phosphorylating the site of Ser318. Furthermore, UBQLN4 interacts with BCL2A1 and BCL2L10 and enhances the drug resistance by stabilizing BCL2A1 and BCL2L10. Our results thus not only revealed lots of potential candidates for ATM but also unveiled a mechanism that a new ATM substrate UBQLN4 regulates apoptosis in mesothelioma by stabilizing BCL2A1 and BCL2L10. Taken together, our data suggest that ATM‐mediated phosphorylation of UBQLN4 at Ser318 may regulate mesothelioma cell apoptosis in response to DNA damage.

Malignant mesothelioma (MM) is a rare neoplasm with poor prognosis, which arises primarily from the surface serosal cells of the pleura, peritoneum, and pericardium and has been classified histologically into three subtypes : epithelioid, sarcomatoid, and biphasic [37]. According to reports, the median survival of patients with mesothelioma is only 8.9–14 months [38, 39]. There are no generally accepted guidelines for radical treatment of MM. For example, the common treatment methods such as radiotherapy and chemotherapy cannot achieve good treatment results. The finding that UBQLN4 is high expressed in mesothelioma and knockdown of UBQLN4 enhances the sensitivity to DNA damage drugs may provide a new molecular strategy for the treatment of malignant mesothelioma.

Ataxia‐telangiectasia mutation (ATM) kinase, as a vital regulator of DNA damage response, plays a significant role in DNA damage, cell metabolism [2], and the cell cycle by phosphorylating an array of downstream substrates [3, 4]. If the damages overweight the repair in genome, ATM could induce programmed cell death by targeting histone H2AX, MDC1, NBS1, CHK2, p53, and MDM2 [8, 9, 10, 11, 12, 13]. In this study, we are the first to use new method to screen substrates for ATM, and the screening result not only accurate but also repeatable, which provides a potential molecular database and a series of new ideas for exploring ATM substrates.

Ubiquilins (Ubqlns) is a type of cytoplasmic protein that exerts protein degradation by enhancing autophagy‐mediated degradation and participating in endoplasmic reticulum‐related protein degradation [40, 41]. UBQLN4 is a member of the ubiquitin family that consists of five known members (ubiquilin 1, ubiquilin 2, ubiquilin 3, ubiquilin 4, and ubiquilin L) and has emerged as a candidate substrate for ATM. Recent studies have shown that UBQLN4 is involved in various intracellular processes such as autophagosome maturation, p21 regulation [27], and motor axon morphogenesis [28]. However, the biological function of UBQLN4 in most tumors is not clear. In this study, we not only confirmed that UBQLN4 is an ATM substrate but also verified that Ser318 is a major phosphorylation site following DNA damage. Furthermore, UBQLN4 inhibits the apoptosis in mesothelioma by stabilizing BCL2A1 and BCL2L10. All of these findings increase our understanding of UBQLN4 in DNA damage. However, how UBQLN4 stabilizes BCL2A1 and BCL2L10 still needs further research and exploration. This could be a new feature and mechanism of the ubiquilin family.

## 5 Conclusions

Taken together, we uncover that about fifty potential substrates for ATM and clarify the mechanism that UBQLN4 inhibits the apoptosis in mesothelioma by phosphorylating the site of Ser318 and stabilizing BCL2A1 and BCL2L10. UBQLN4 may be of great therapeutic potential for targeting mesothelioma and other diseases. Other potential substrates of ATM need be further explored to clarify the network of ATM and may act as a potential therapeutic target or provide new therapeutic strategies for human diseases.

## Conflict of interest

The authors declare that they have no conflict of interest.

### Peer Review

The peer review history for this article is available at https://publons.com/publon/10.1002/1878‐0261.13058.

## Author contributions

FL, HYD, and LLG designed the experiments. FL, RSP, and HYD performed most of the experiments. LLG, YY, CYL, YJX, and HC performed part of the experiments. FL, RSP, and HYD prepared and wrote the manuscript. XYZ, YJN, and RGH reviewed and edited the manuscript. All authors discussed the results and commented on the manuscript.

## Supporting information


**Table S1**. GFP competition results of potential ATM substrate candidates.

**Table S2**. shRNAs sequence.

**Table S3**. DNA damaging drugs used in this study.

**Table S4**. The information of patient.

## Data Availability

All experimental data during this research are included in the published article and its supplementary files. The datasets and materials in this study are available on reasonable request from corresponding authors or first author.
