# Supplementary material for: UBQLN4 is an ATM substrate that stabilizes the anti‐apoptotic proteins BCL2A1 and BCL2L10 in mesothelioma
Source: Mol Oncol. 2021 Aug 30;15(12):3738–52. doi: 10.1002/1878-0261.13058 (PMC8637560; doi:10.1002/1878-0261.13058)
Supplement: Supplementary file 1 — Table S1. GFP competition results of potential ATM substrate candidates. [file MOL2-15-3738-s003.docx]

**Table 1 GFP competition results of potential ATM substrate candidates.**

| Gene | CISP | CPT | DOX | MTX | Gene | CISP | CPT | DOX | MTX |
| --- | --- | --- | --- | --- | --- | --- | --- | --- | --- |
| Adnp | -0.181 | -0.124 | -0.197 | -0.077 | Phf6 | 0.219 | -0.071 | 1.232 | -0.051 |
| Arid2 | -0.016 | 1.149 | 0.655 | 1.342 | Phrf1 | -1.127 | -0.826 | -1.121 | -0.338 |
| Atrx | -0.499 | -0.806 | -1.218 | 1.384 | Psip1 | -1.100 | -1.439 | -0.853 | -0.546 |
| Bap1 | -1.184 | -1.084 | -1.205 | -0.420 | Rsf1 | -0.579 | -0.779 | -1.398 | 0.064 |
| Baz2a | -1.254 | -1.330 | -0.344 | -0.709 | Sas10 | -0.505 | -0.127 | -0.452 | -0.814 |
| Bcl7a | -0.399 | -0.038 | -0.229 | 0.102 | Scaf11 | 0.904 | 1.150 | 0.870 | 0.740 |
| Brwd3 | -0.315 | -0.454 | 0.053 | -0.021 | Sf3a1 | -0.580 | -0.502 | -0.719 | 0.294 |
| Ccdc16 | -0.426 | -0.309 | -0.323 | 0.076 | Sf3b1 | -0.430 | -0.242 | -1.167 | 0.294 |
| Clasp | -1.095 | -0.648 | -1.469 | -0.533 | Smarcad1 | -0.247 | -0.432 | -0.224 | -0.168 |
| Ddx24 | -0.886 | -0.662 | -0.781 | -0.781 | Snip1 | 0.327 | -0.379 | -0.160 | -0.125 |
| Fbxl11 | -0.354 | 0.155 | -0.060 | 0.219 | Srrm2 | 0.056 | -0.133 | -0.038 | 0.102 |
| Foxk1 | -0.684 | 0.234 | -0.434 | 0.173 | Tcf12 | -0.315 | 0.114 | -0.036 | -0.538 |
| Foxk2 | -1.513 | -1.549 | -0.809 | -0.307 | Tcof1 | -1.196 | -1.473 | -1.280 | -0.535 |
| Hmga | -0.252 | 0.204 | 0.130 | -0.048 | Thoc2 | -1.489 | -0.615 | 0.082 | -0.396 |
| Hsp90ab1 | -1.308 | -1.081 | -1.475 | 0.815 | Thoc5 | -1.019 | -1.058 | -0.218 | -0.694 |
| Khrsp | -0.053 | 0.281 | -0.142 | 0.247 | Tpx2 | -0.310 | -0.471 | -0.448 | -0.273 |
| Larp1 | -1.112 | -2.440 | -0.485 | -2.441 | Trip12 | -1.258 | -1.553 | -1.220 | -1.016 |
| Map1b | -1.084 | -1.444 | -1.837 | -0.638 | Ubap2l | -0.600 | -0.742 | -0.824 | -0.682 |
| Mcm6 | -0.781 | -1.076 | -0.951 | -0.845 | Ubqln4 | -0.734 | -1.262 | -0.952 | -0.133 |
| Myh10 | -0.514 | -0.842 | -0.745 | -0.018 | Ubr4 | -0.241 | 0.381 | 0.538 | 0.310 |
| Nipbl | -1.052 | -0.964 | -1.409 | -1.257 | Ubr5 | -0.938 | -1.785 | -1.521 | -0.135 |
| Nkap | -0.259 | 0.184 | -0.060 | 0.072 | Wtap | -0.246 | 0.012 | 0.140 | -0.164 |
| Npat | 0.054 | -0.109 | -0.295 | 0.104 | Zc3h13 | 0.143 | 0.622 | -1.058 | -0.869 |
| Numa | -0.195 | -0.369 | 0.055 | -0.343 | Zc3h14 | -0.548 | -0.626 | -0.700 | -0.444 |
| Ogfr | 0.342 | 0.320 | 0.359 | 0.209 | Zc3h4 | -0.609 | -0.328 | -0.873 | -0.216 |
| Pcm1 | -1.467 | -0.874 | -0.137 | -0.220 | Zranb2 | -0.097 | -0.397 | 0.055 | -0.243 |

Log_2_RI was shown in the table, numbers less than 0 indicate drug sensitivity, numbers greater than 0 indicate drug resistance.
