# Supplementary material for: UBQLN4 is an ATM substrate that stabilizes the anti‐apoptotic proteins BCL2A1 and BCL2L10 in mesothelioma
Source: Mol Oncol. 2021 Aug 30;15(12):3738–52. doi: 10.1002/1878-0261.13058 (PMC8637560; doi:10.1002/1878-0261.13058)
Supplement: Supplementary file 3 — Table S3. DNA damaging drugs used in this study. [file MOL2-15-3738-s001.docx]

**Table 3 DNA damaging drugs used in this study**

| Abbreviation | Drug Full name | Mechanism of Action |
| --- | --- | --- |
| DOX | Doxorubicin | DNA topoisomerase II inhibitor |
| CPT | Camptothecin | DNA topoisomerase I inhibitor |
| CISP | Cisplatin | DNA intra-strand crosslinking |
| MTX | Methotrexate | Dihydrofolate reductase (DHFR) inhibitor |
